# Quantifying Clonal and Subclonal Passenger Mutations in Cancer Evolution

**DOI:** 10.1371/journal.pcbi.1004731

**Published:** 2016-02-01

**Authors:** Ivana Bozic, Jeffrey M. Gerold, Martin A. Nowak

**Affiliations:** 1 Program for Evolutionary Dynamics, Harvard University, Cambridge, Massachusetts, United States of America; 2 Department of Mathematics, Harvard University, Cambridge, Massachusetts, United States of America; 3 Department of Organismic and Evolutionary Biology, Harvard University, Cambridge, Massachusetts, United States of America; National Research Council of Canada, CANADA

## Abstract

The vast majority of mutations in the exome of cancer cells are passengers, which do not affect the reproductive rate of the cell. Passengers can provide important information about the evolutionary history of an individual cancer, and serve as a molecular clock. Passengers can also become targets for immunotherapy or confer resistance to treatment. We study the stochastic expansion of a population of cancer cells describing the growth of primary tumors or metastatic lesions. We first analyze the process by looking forward in time and calculate the fixation probabilities and frequencies of successive passenger mutations ordered by their time of appearance. We compute the likelihood of specific evolutionary trees, thereby informing the phylogenetic reconstruction of cancer evolution in individual patients. Next, we derive results looking backward in time: for a given subclonal mutation we estimate the number of cancer cells that were present at the time when that mutation arose. We derive exact formulas for the expected numbers of subclonal mutations of any frequency. Fitting this formula to cancer sequencing data leads to an estimate for the ratio of birth and death rates of cancer cells during the early stages of clonal expansion.

## Author Summary

Cancer is the consequence of an evolutionary process, which lasts several decades, is impossible to observe during most of its time, and only becomes apparent in late stages. We use mathematical modeling to shed light on the evolutionary dynamics of cancer by studying the accumulation of passenger mutations. We show that the frequencies obtained by passenger mutations depend strongly on the ratio of death and birth rates of cancer cells. We use genetic data of colorectal cancer to estimate this important quantity in vivo. We estimate the size of the cancer cell population that was present when a specific mutation first emerged. Our theory informs the analysis of cancer sequencing data and the phylogenetic reconstruction of cancer evolution.

## Introduction

In healthy tissues, cell division and cell death are tightly controlled processes, which enable a precise balance assuring that the number of cells in the body remains approximately constant. However, during each cell division mistakes in DNA replication can occur, leading to accumulation of mutations in individual cells [1, 2]. The majority of such mutations are effectively neutral (passengers), but some of them (drivers) can provide selective advantage to the cell, by tipping the balance of cell division and death slightly in favor of increased proliferation [3–5]. This unwanted evolution [6–8] of somatic cells can lead to a clonal expansion of cells with driver mutations, which can ultimately result in the formation of tumors and seeding of new lesions in distant tissues [9–11].

Sequencing efforts over the past decades have resulted in a compendium of genetic alterations in the exomes of common human cancers and revealed that adult cancers harbor dozens (leukemias) to hundreds (lung cancer and melanoma) of somatic mutations. A typical tumor is thought to contain 2-8 driver gene mutations, with the rest being neutral passengers [9]. Unlike driver mutations, passengers cannot be attacked by conventional targeted therapy, but some of them can become targets for immunotherapy or induce resistance to treatment [12–14]. In addition, passenger mutations can provide information about the timing of cancer evolution in individual patients by acting as a molecular clock [15].

Recent studies found that the evolution of metastases in colorectal [16] and pancreatic cancer [15], and even the evolution of primary colorectal cancer follows largely neutral evolution [17], in which the founding cell starts a clonal expansion during which cells accumulate neutral mutations (passengers) rather than drivers. Here we study neutral evolution during clonal expansion and show that passenger mutations can be used to infer the parameters of the tumorigenic process. Our model is a generalization of the famous Luria-Delbrück model for studying resistance mutations in bacteria [18, 19]. In contrast to the original Luria-Delbrück model, in which wild type and mutant populations grew deterministically and mutation occurred stochastically, in our model all cell types grow stochastically; our model also includes cell death. Many authors have studied the fully stochastic version of the Luria-Delbrück model in which all mutants are treated as a single population [20–25]. In these models there are only two cell populations: wild type and mutant. In contrast, here we study populations started by individual passenger mutations separately.

## Results

We model the accumulation of passenger mutations during clonal expansion of cancer using a multi-type branching process [26, 27] that starts with a single type-0 cell. All cells in the process divide with rate *b* and die with rate *d*. At each division, one of the daughter cells receives a new passenger mutation with probability *u*, which starts a new type. We are interested in the mutations accumulating in the exome during tumor evolution, so we are mostly interested in the mutation rate *u* = 0.015, which is the product of the normal point mutation rate per cell division (∼5 ⋅ 10^−10^) and the number of base pairs in the exome (∼3 ⋅ 10^7^) [5].

**Fig 1:**
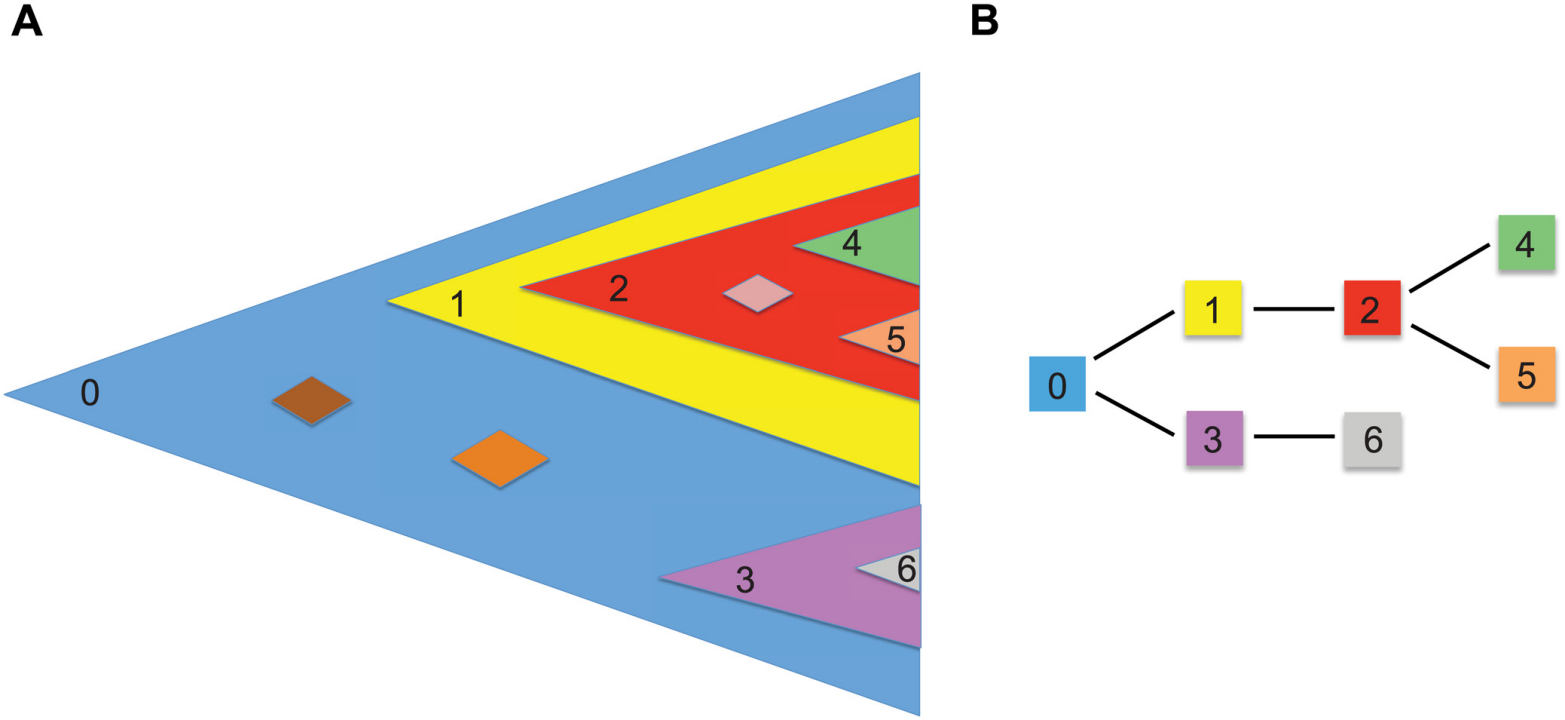
Evolutionary dynamics of passenger mutations during clonal expansion. (A) New passenger mutations can be lost due to stochastic drift (diamonds). Successful mutations form surviving lineages. We order successful mutations by their time of appearance. Individual cells can harbor many passenger mutations and various different phylogenies can arise (B). In the example shown, mutation 2 appears in a cell that already harbors mutation 1. Thus all cells that have mutation 2 also have mutation 1. Similarly, all cells cells that have mutations 4 or 5 also harbor mutations 1 and 2. Mutation 3 forms an independent clone. We calculate the likelihood of different phylogenies and the expected number of subclonal mutations of any frequency.

Any new mutation that appears in the population can be lost due to stochastic fluctuations. The probability that its lineage will not survive is *δ* = *d*/*b*, the ratio of the death and the birth rates [26], and we will see later that the limiting behavior of the process is strongly dependent on *δ* and not the individual values of *b* and *d*. We label the mutations with surviving lineages (“successful” mutations) according to their order of appearance. We are interested in the fraction of cells harboring mutation *k*, for *k* ≥ 1, and the phylogenetic relationships [28] between first appearing successful mutations (Fig 1).

Throughout the paper we mostly assume that the birth rate is *b* = 0.25 per day, a typical value for colorectal cancer [5], but all results scale accordingly for other values of *b*. The ratio of death and birth rates in cancer has been estimated to be on the order of *δ* = 0.72 in fast-growing colorectal cancer metastases [29] to *δ* = 0.99 in early tumors [5], and we focus on *δ* values in this range.

The average number of passenger mutations with surviving lineage that are present in a population of *M* cells is *Mu*[14]. For a tumor containing *M* = 10^9^ cells (∼1 cm in diameter) the number of passenger mutations is ∼1.5 ⋅ 10^7^. The number of mutations is thus almost half of the length of the exome, but the vast majority of those mutations are present in a small number of cells, far below the detection limit of current sequencing technologies [30]. We are mostly interested in the mutations present in a sizable fraction of the population (above 0.1% of all cells). We assume an infinite allele model, in which each mutation can appear only once.

We perform Monte Carlo simulations of the multitype branching process using the Gillespie algorithm [31]. Between 5,000 and 10,000 surviving runs are used for each parameter combination.

### Probability of fixation of new mutations

In a pure birth process, *d* = 0, the founding cell (type-0 and no other mutations) is always present in the population, and thus all new mutations are subclonal; they are present in less than 100% of tumor cells. However, with death rate *d* > 0, new mutations appearing during clonal expansion can reach fixation in the population. We show in Methods that the probability that the *k*-th mutation with surviving lineage eventually fixates and becomes present in all cells is given by

$$\rho_{k}\approx {\left(\frac{u}{u-\log\delta }\right)}^{k}.$$ (1)

If the *k*-th surviving mutation reaches fixation, it is implied that all preceding *k* − 1 surviving mutations (labeled 1 to *k* − 1) also reach fixation. Therefore each cell in the lesion has the first *k* mutations.

From formula (1) we see that the probability of fixation increases with both the mutation rate, *u*, and the death-birth ratio, *δ*. Assuming normal mutation rate in the exome we have *u* = 0.015. Thus, in a fast-growing population, in which *δ* is significantly smaller than 1, it is unlikely that any new mutation reaches fixation. For example, for *δ* = 0.72, the probability that the first appearing mutation with surviving lineage reaches fixation is *ρ*_1_ ≈ 0.04. For *δ* = 0.96 this probability is *ρ*_1_ ≈ 0.27. When growth is particularly slow, for example *δ* = 0.99, the first mutation with surviving lineage has a *ρ*_1_ ≈ 0.6 chance of fixation, while the second has a *ρ*_2_ ≈ 0.36 chance and even the fifth can fixate with probability *ρ*_5_ ≈ 0.08.

### Frequency and phylogenies

**Fig 2:**
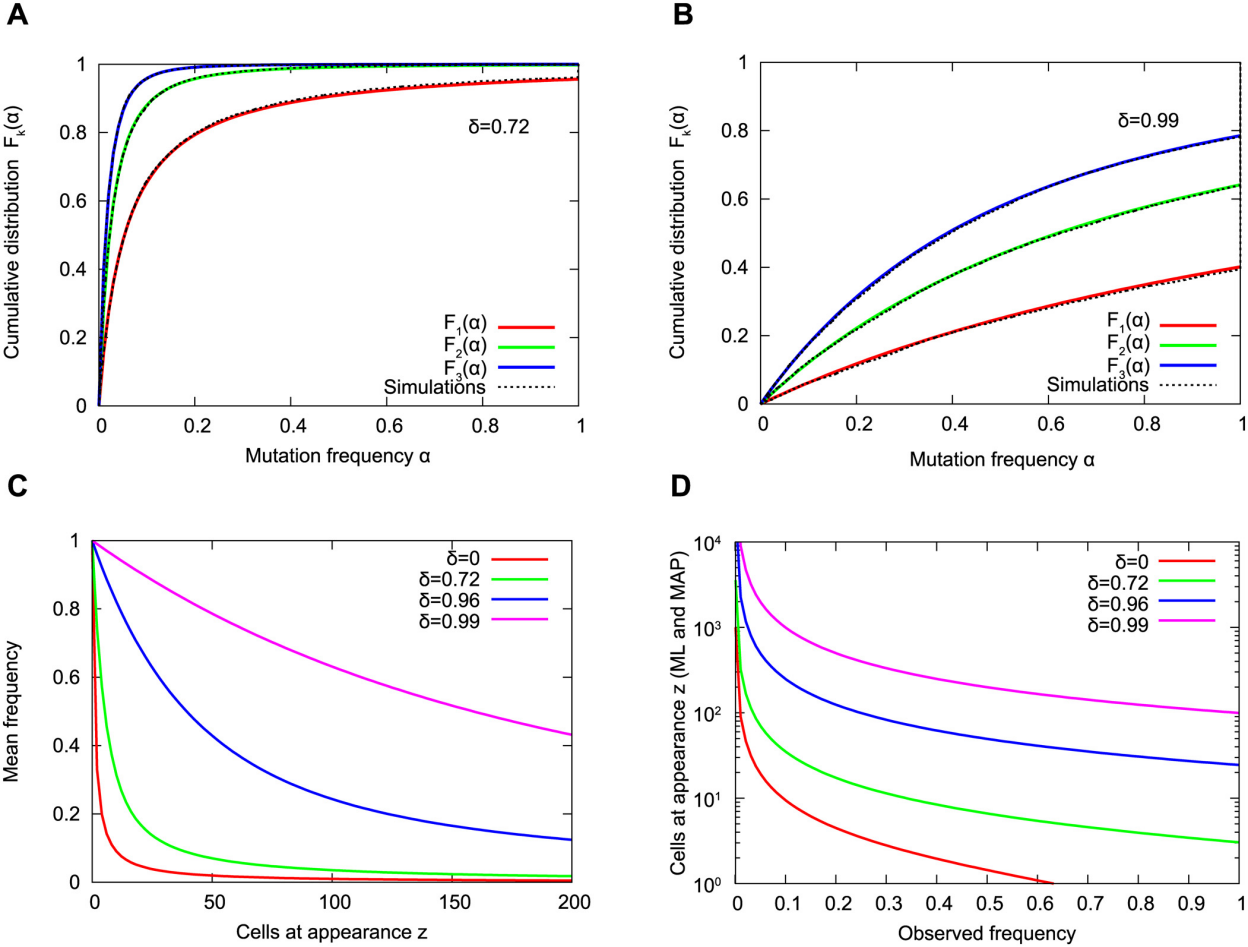
Frequency of passenger mutations. (A-B) Cumulative distribution function for the first three successful mutations. The y-axis shows the probability that the mutation has a frequency of less than *α*. Comparison between formula (2) and exact computer simulations of the stochastic process with death-birth ratios *δ* = 0.72 (A) and *δ* = 0.99 (B). For *δ* = 0.72, the median frequencies of the first three successful mutations are below 5%. For *δ* = 0.99, they are all above 40%. (C-D) Mutation frequency versus time of appearance. (C) Mean frequency attained by a mutation which arose when there were *z* other cells in the population, for different values of the death-birth ratio, *δ*. (D) Maximum likelihood and maximum a posteriori estimate (which are approximately equal) for the number of cells in the population when the mutation with frequency *α* arose. Passenger mutation rate *u* = 0.015 (product of the number of basepairs in the exome, *L* ∼ 3 ⋅ 10^7^, and the normal point mutation rate during cell division, *μ* ∼ 5 ⋅ 10^−10^).

We show in Methods that the cumulative distribution function for the frequency of cells with the *k*-th mutation is given by

$$F_{k}\left(\alpha \right)\approx 1-{\left(\frac{u}{u-\log[1-\alpha (1-\delta )]}\right)}^{k}$$ (2)

for 0 < *α* < 1. Formula (2) is the probability that the frequency of cells carrying the *k*-th mutation is less than *α*. Note that *F*_*k*_(*α*) does not approach 1 as *α* = 1 due to a non-zero fixation probability. The excellent agreement between formula (2) and exact computer simulations of the stochastic process is shown in Fig 2A and 2B. The fixation probability of the *k*-th mutation is precisely *ρ*_*k*_ = 1 − *F*_*k*_(1). For fast growing tumors, *δ* = 0.72, the median frequencies of the first three surviving mutations are all smaller than 5% (Fig 2A). In contrast, for slow growing tumors, *δ* = 0.99, the median frequencies of the first three mutations are all greater than 40% (Fig 2B).

**Fig 3:**
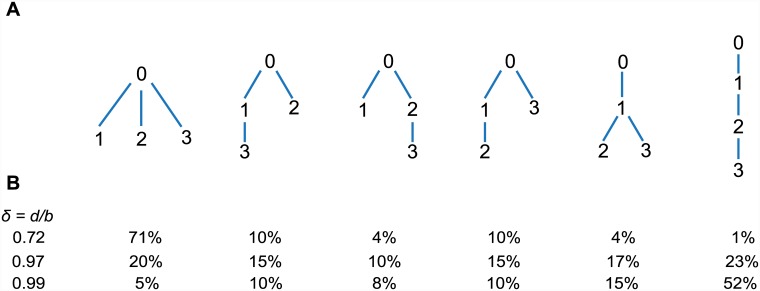
Likelihood of phylogenetic trees. (A) All six phylogenetic trees containing the first three surviving passenger mutations are shown. (B) Probabilities of each tree for different values of the death-birth ratio, *δ* (formulas shown in Methods). For *δ* = 0.72, the first tree is the most likely. For *δ* = 0.99, the sixth tree is the most likely. For intermediate *δ* = 0.97, the most likely tree shape is that of trees 2-4. Passenger mutation rate *u* = 0.015.

Another significant difference between slow growing and fast growing tumors is exhibited by the phylogenetic relationships among the first surviving mutations. When *δ* = 0.72, the most likely phylogeny including the founding population (type-0) and the first surviving mutations is star-like (first tree in Fig 3A). In contrast, when *δ* = 0.99, the most likely phylogeny is linear (last tree in Fig 3A).

Formulas for the probabilities of all six phylogenetic trees involving the founding population and the first three surviving mutations are given in Methods. In Fig 3 we plot all six trees and their probabilities for various values of *δ*. For all values of *δ*, either the first or the last tree are the most likely. However, if we do not possess the knowledge of the order in which the first mutations appeared, then trees 2-4 (Fig 3A) are indistinguishable, and for intermediate *δ* (i.e. *δ* = 0.97), the shape of trees 2-4 will be the most likely.

### Frequency and time of appearance

Let us now assume that there were *z* other cells in the population when a certain mutation (with surviving lineage) appeared. In Methods we calculate the probability distribution for the eventual frequency of that mutation. We show that the expected frequency that the mutant eventually achieves is

$$E\left(x\right)=\frac{1-\delta^{z+1}}{\left(1-\delta )(z+1\right)}.$$ (3)

If *δ* = 0, which means no cell death, *d* = 0, the expected frequency is 1/(*z* + 1). However, for *δ* > 0 and especially when *δ* is close to 1, which is the most relevant case for cancer growth, the expected frequency is much higher than 1/(*z* + 1). For example, consider a mutation (with surviving lineage) which appears when there are 100 other cells present in the tumor; for *δ* = 0 this mutations reaches an expected frequency of *E*(*x*) = 0.01; for *δ* = 0.99 it reaches an expected frequency of *E*(*x*) = 0.63 (Fig 2C).

So far we have studied the branching process by looking forward in time. We now derive several results that are useful for inferring knowledge about the early evolutionary history of the tumor obtained from data at late stages of its evolution. To start, we ask an inverse question: if a mutation is present at frequency *α*, when did it first appear? We show in Methods that the maximum likelihood and maximum a posteriori estimates for the number of cancer cells that were present at the time when that mutation arose are approximately the same and given by

$${\hat{z}}_{\text{MAP}}\approx {\hat{z}}_{\text{ML}}=-\frac{1}{\log[1-\alpha (1-\delta )]}.$$ (4)

Note that the estimated number of cells at appearance increases with *δ* (Fig 2D).

We see from formula (4) that a mutation that is present in 10% of the population has most likely appeared when there were as few as 10 cells (if *δ* = 0 which is unlikely in cancer) to as many as 1000 cells (if *δ* = 0.99). Similarly, a mutation that is present in 50% of cells most likely appeared when there were as few as 1 other cell (if *δ* = 0) to as many as 200 cells (if *δ* = 0.99).

### Expected number of clonal and subclonal mutations

We prove in Methods that the expected number of subclonal mutations present at a frequency larger than *α* is

$${\bar{m}}_{s}=\frac{u(1-\alpha )}{(1-\delta )\alpha }$$ (5)

Similarly, the expected number of clonal passenger mutations is given by

$${\bar{m}}_{c}=\frac{\delta u}{1-\delta }$$ (6)

The number of sublclonal mutations is highly dependent on the ratio *δ*. When there is no cell death, *δ* = 0, there is on average only a single passenger mutation that is present at a frequency higher than 1%. On the other hand, if *δ* = 0.99, there will be about 150 passenger mutations present in more than 1% of cancer cells (Table 1).

**Table 1:** Expected number of subclonal and clonal mutations for different values of *δ* = *d*/*b*.

***δ***	> 0.1%	> 1%	> 10%	> 50%	Clonal
0	15.0	1.5	0.14	0.015	0
0.72	53.5	5.3	0.48	0.05	0.04
0.96	374.6	37.1	3.37	0.38	0.36
0.99	1498.5	148.5	13.5	1.5	1.48
0.999	14985	1485	135	15	15

Values calculated using formulas (5) and (6). We assumed normal point mutation rate in the exome *u* = 0.015.

Formula (5) can be fitted to cancer sequencing data to determine how well the branching process model of neutral evolution describes the observed mutation frequencies and to extract the most likely parameters of the process. We fit our formula to the TCGA (http://cancergenome.nih.gov/) colorectal cancer dataset, publicly available at https://dcc.icgc.org/releases/current/Projects/COAD-US. All samples were classified as either microsatellite-stable (MSS) or instable (MSI) based on the sample’s total number of mutations [32], and their purity and ploidy have been assessed [33]. We required samples with ploidy between 1.8 and 2.2 so that the cancer was not too far from diploid and chromosomal instability and LOH did not significantly alter the distribution of allele frequencies. We further required a purity estimate of at least 70%. A total of 42 samples passed both of these criteria and were fit to our formula, after adjusting their allele frequency to account for sample purity.

**Fig 4:**
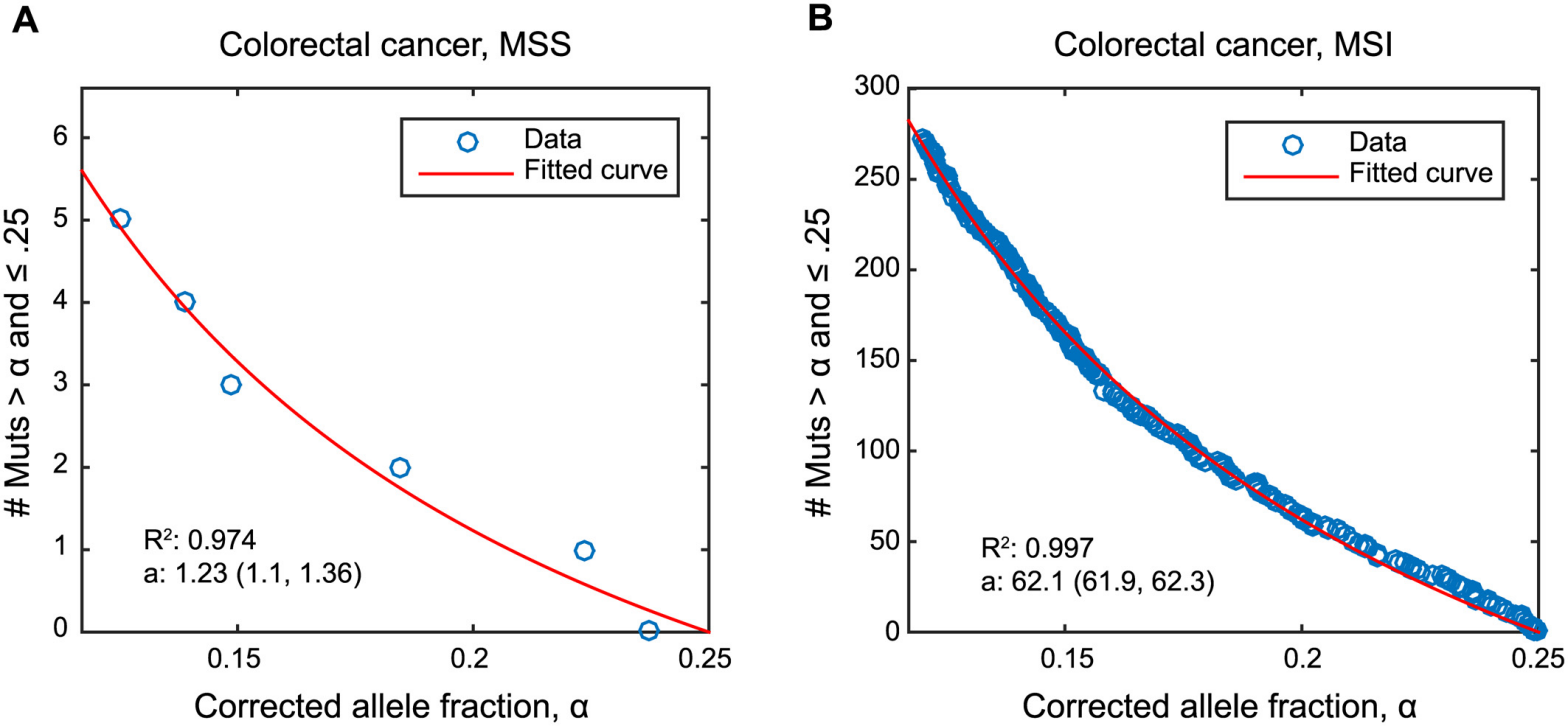
Predicted and observed numbers of subclonal mutations in colorectal cancer. Exome sequencing data for two colorectal cancers from the TCGA dataset, (A) microsatellite stable (MSS) and (B) microsatellite instable (MSI), show the corrected allele fraction of each detected mutation (observed allele fraction divided by purity). Mutations with allele frequency of 25% or more may be clonal [34] and mutations with corrected allele frequency below 12% can be difficult to detect reliably. Thus we focus on mutations with fractions between 0.12 and 0.25, and plot the number of mutations with fraction between *α* and 0.25 as a function of *α*. The data are fit to the formula for the number of mutations with the corresponding allele frequency Eq (7). The best fit for $a=\frac{u}{2(1-\delta )}$ and its corresponding 95% confidence interval is shown for each sample.

In Fig 4 we plot the number of mutations with allele fraction between 0.12 and 0.25, found in two colorectal cancer samples from the TCGA dataset. Mutations with allele frequency of 25% or more may be clonal (for example, a heterozygous mutation present in one copy of a tetraploid chromosome). On the other hand, mutations with allele frequency below 10% can be difficult to detect (which translates to 12% corrected frequency as the average purity of our samples is 85%). In [34], the authors fit the same data to a formula they derived using a deterministic approximation with no cell death.

Assuming that there is no loss of heterozygosity and that all mutations are present in a single allele of a diploid cell, the allele frequency of a mutation is 1/2 of its cancer cell frequency. It follows from formula (5) that the number of mutations with allele frequency larger than *α* but smaller than 0.25 is given by

$$\frac{u}{2(1-\delta )}\left(\frac{1}{\alpha }-\frac{1}{0.25}\right).$$ (7)

Out of the 42 colorectal cancer samples that passed our filtering criteria, 16 had fits with with *R* ^2^ ≥ 0.9, and we show them in S1 Fig. For the two cancers in Fig 4, the best fit is obtained for *a* = *u*/(1 − *δ*) = 1.23 (Fig 4A) and *a* = 62.1 (Fig 4B). More generally, the median value for *a* in MSS cancers is 2.86, and 27.61 for MSI cancers. For MSS cancers, assuming a normal passenger mutation rate in the exome *u* = 0.015 leads to birth-death ratio *δ* = 0.997. This value is between the estimates of net proliferation rates in premalignant colorectal tumors (*δ* ∼ 0.999) and colorectal cancers (*δ* ∼ 0.99) obtained from cancer incidence data [35]. If the mutation rate is elevated 10-fold, then the best fit is obtained with death-birth ratio *δ* = 0.97. Note that fitting formula (7) and assuming death rate *d* = 0 (which implies *δ* = 0) to the data means that the mutation rate during tumor evolution needs to be ∼400 fold higher than the normal mutation rate, which is unlikely.

Interestingly, for *δ* close to 1, the number of subclonal mutations with frequency above 50% is approximately equal to the number of clonal passengers collected during tumor progression. Thus subtracting the number of subclonal mutations with frequency above 50% from the number of all clonal mutations in the cancer will be an estimate for the number of clonal mutations present in the first malignant cell.

## Discussion

In summary, we have shown that the frequencies of the first and thus most abundant passenger mutations are influenced not only by the mutation rate, but also by the death-birth ratio, *δ* = *d*/*b*, of the cancer cells. If *δ* is close to 1, which is the relevant case for slow overall growth, then several clonal passengers may not have been present in the first tumor cell, but were collected during clonal expansion. The ratio *δ* also determines the shape of the cancer’s phylogenetic tree, which is star-shaped for fast growth and linear for slow growth (Fig 3). Additionally, if we consider a mutation that has a certain observed frequency in the population of cancer cells, we can ask: how many cells were present when that mutation arose? The answer varies hundred-fold as the death-birth ratio, *δ*, changes from 0 to 0.99. This is particularly relevant as high levels of cell death are reported in both malignant and premalignant tissues [36, 37].

In this work, we derive a simple form Eq (7) for the cumulative distribution of mutant allele frequencies under a neutral model of cancer evolution. Fitting the cumulative distribution of allele fractions in a sample to this functional form and analyzing the goodness of fit provides information about the nature of the process underlying the generation of mutant alleles. We computed this distribution with data from each patient by first dividing all allele fractions in a single patient by the sample purity and then restricting our view to allele fractions in the interval [.12,.25]. The high end of interval was chosen to minimize the chance clonal mutations appeared in it and the low end was chosen to ensure that sequencing was powered to detect such mutations. The cumulative distribution of allele fractions in this interval was computed at each mutant allele fraction as the number of mutations greater than that fraction but less than or equal to the cutoff at 0.25. Finally, we performed a least-squares fit to the functional form described using MATLAB and report fits with *R* ^2^ values at least 0.9.

Our model is applicable to individual cancers in which there are no subclonal drivers at observable cell frequencies. This includes both liquid and solid tumors. It has recently been shown that colorectal tumors fit this model often [17, 34]. In contrast, some liquid cancers such as chronic lymphocytic leukemia usually harbor subclonal drivers, and are thus not good candidates for the application of our model [38].

In a previous paper [14], we have studied the accumulation of individual resistance mutations in cancer using a fully stochastic Luria-Delbrück model. For targeted therapies, resistance mutations are estimated to be rare: only about a hundred positions in the genome can give rise to resistance if mutated [29, 39, 40]. Therefore all results in Ref. [14] were derived in the limit of very small mutation rates, about ∼10^−7^ per cell division. In that scenario, two resistance mutations are virtually never present in the same cell, and the fixation of mutations in the population is extremely unlikely. In contrast, here we are interested in the accumulation of passenger mutations in the whole exome, which leads to a large number of passenger mutations in the population. Even individual cells contain many different mutations. Therefore, new questions arise and a new mathematical approach is needed.

Our model is a continuous time version of the infinite alleles branching process introduced by Griffiths and Pakes [41], and is a special case (birth-death process) of the model studied recently by Wu and Kimmel [42]. These works were mostly interested in the limiting frequency spectrum of the process, namely the number of mutations present in *j* individuals as time *t* → ∞; for example, Wu and Kimmel provide an explicit expression for the mean limiting frequency spectrum for the birth-death process in terms of the hypergeometric function. We study the same process with respect to tumor size and derive explicit expressions for the frequencies of mutations according to their order of appearance. We also study the expected number of mutations above a certain frequency, which has connections to the frequency spectrum.

Sottoriva and Graham [34] estimate the number of cells, including the new cell, that were present when a mutation with observed frequency *α* appeared by 1/*α*, using a deterministic model with no cell death. A deterministic model is always useful as it provides the simplest approach to study evolutionary dynamics. Our formula (4) provides the stochastic correction to their prediction.

Recently, Durrett [43] derived formulas for the expected number of passenger mutations present at a frequency larger than *α*. His process differs from ours as in his model mutations occur independent of cell division. Consequently, the founding cell can collect mutations prior to the first cell division, and even for *d* = 0 there could be passenger mutations that are clonal. In contrast, in our model for *d* = 0 all mutations are strictly subclonal.

In this paper we do not consider loss of heterozygosity (LOH), which implies that mutations can be lost from the cell during cell division. The rate of LOH is on the order of 10^−6^ or lower per cell division in tumors that do not have chromosomal instability (CIN) [44]. For such small LOH rate, if LOH events are neutral or deleterious, the fraction of cells that gained a mutation but then lost it will be very small, and our results would still hold; our results would also hold for LOH events that occur at higher rates but are deleterious. In a subsequent paper we will study the effect of LOH events on the evolutionary dynamics of passenger mutations, and focus on situations where they occur with high frequency (CIN) [45, 46].

Similarly, here we do not consider the effect of new driver mutations that may appear during clonal expansion of cancer [5, 47–49]. The addition of drivers may change both the observed mutational frequencies and the phylogenies of the occurring mutations. Instead we focus only on the accumulation of neutral mutations, which is the relevant case for studying the growth of metastases and even some primary tumors [15–17].

While cancer spends much time in clonal expansion, plateau stages are also common. The effects of plateau stages after clonal expansion on the frequency of passenger mutations can be studied using a density-dependent branching process used previously in the context of resistance to cancer therapy [50]. This density-dependent model can be approximated analytically with a two phase model: a branching process with constant birth and death rates *b* and *d* corresponding to the growth phase, and a plateau phase of length *T* in which birth rate of all cells is approximately equal to *d*. In this model, mutations present at the end of the plateau phase can be either “old” mutations that were present at the end of the growth phase, or “new” mutations that appeared during the plateau phase and were not lost. In the large time *T* limit, all old mutations will either reach fixation or be lost in the population, but for shorter times *T* frequencies of old mutations can be approximated by their frequencies at the end of the exponential growth phase. On the other hand, the number of new mutations that are present above a certain frequency can be studied analytically using techniques from [50] to show that new mutations will in general not be present at observable frequencies if the population size at the plateau is on the order of millions of cells or higher. Rodrigues-Brenes et al. recently studied inhibited cancer growth in the context of stem-cell driven cancers [51].

In our model we do not explicitly take into account the possible existence of a differentiation hierarchy within the cancer population: namely, the existence of cancer stem cells, which are able to propagate the tumor population indefinitely, and differentiated cells, which have limited life-spans. However, our results can also inform the study of this more complicated situation, and our analysis in fact applies to cancer stem cells. To add differentiated cells, we can consider the model in which, in addition to our basic assumptions, stem cells can also divide to produce one stem cell and one differentiated cell of generation 1, differentiated cells of generation *i* divide to produce two differentiated cells of generation *i* + 1 and the lifespan of differentiated cells is *n* divisions (i.e. differentiated cells of generation *n* are lost from the population). A reasonable estimate for the number of divisions before mitotic arrest is *n* = 10 (e.g. 4-6 divisions in colon [52] and 15-20 in hematopoietic system [53]), which means that each differentiated cell of generation 1 produces ∼1000 cells before they are lost from the population. Typical detectable tumors contain billions of cells; mutations that occur in the lineage of a single differentiated cell will remain confined to that lineage, which will contain no more than ∼1000 cells (or no more than ∼10^6^ cells if *n* = 20), and will not reach a significant fraction in the population (less than 1/10^6^ for *n* = 10 or less than 1/10^3^ for *n* as high as 20). Hence mutations appearing in the lineages of differentiated cells will not be present at frequencies above 0.1% or 1% that we are interested in—mutations above these frequencies will be only those in the stem cell population, which will behave as described in our model. The only adjustment that needs to be made to our results when referring to cancer stem cells is the adjustment of the mutation rate to account also for mutations that occur to stem cells during asymmetric divisions.

We recently developed a spatial version of the model studied in this paper, which we mostly analyzed through computer simulations [54]. In this spatial model, tumor growth occurs on a 3-dimensional lattice and birth rate is reduced in the presence of many neighboring cancer cells. This results in the inside of the tumor being in the state of equilibrium between birth and death, while the surface of the tumor is able to expand. If the effective birth rate of cells in the surface of the spatial model is comparable to the birth rate in the well-mixed model, there will be more mutations present above a certain frequency in the spatial case, as the spatial tumor experienced more divisions to reach the same size. However, the addition of migration of tumor cells allows cells to explore less crowded spatial positions and in turn reduces the number of divisions needed to reach the same size as well as the number of mutations above a certain frequency, bringing this model closer to the model without space [54].

## Methods

### Eventual fraction and time of appearance

We are interested in the eventual fraction of cells carrying a successful mutation, which appeared when there were *z* other cells in the population. Let *Y* be the population started by these *z* cells (i.e. cells without the mutation) and *X* the population carrying the mutation. The probability that exactly *i* out of *z* non-mutant cells have surviving lineage is

$$\pi_{i}=\left(\begin{array}{c} z\\ i \end{array}\right){\left(1-\delta \right)}^{i}\delta^{z-i}.$$ (8)

When *i* = 0, the non-mutant fraction dies out and the eventual fraction of the mutant is 1. For *i* ≥ 1, the number of non-mutant cells *Y* ≈ *e* ^(*b*−*d*)*t*^(*V*_1_ + … + *V*_*i*_), where *V*_1_, …, *V*_*i*_ are independent exponentially distributed random variables with mean *b*/(*b*−*d*) [14, 49] and time *t* is large and measured from the time of appearance of the mutant. In other words, the number of cells without the mutation is given by

$$Y=\left\{\begin{array}{cc} e^{(b-d)t}\left(V_{1}+\cdots +V_{i}\right)\;\;\;\; & \text{with}\;\;\text{probability}\;\;\pi_{i},\;\;i\geq 1\\ 0\;\;\;\; & \text{with}\;\;\text{probability}\;\;\pi_{0} \end{array}\right.$$ (9)

Similarly, *X* ≈ *e* ^(*b*−*d*)*t*^ *V*, where *V* is again exponentially distributed random variable with mean *b*/(*b*−*d*). Thus when the number of non-mutant cells with surviving lineage *i* > 0, the eventual fraction of cells with the mutation is

$$x=\frac{X}{X+Y}=\frac{V}{V+V_{1}+\cdots +V_{i}}=\beta \left[1,i\right],$$ (10)

where *β*[1, *i*] is a beta-distributed random variable with probability density function *i*(1 − *w*)^*i*−1^[14]. This allows us to calculate the probability that the fraction of the population carrying the mutation is smaller than *α*, for 0 < *α* < 1:

$$\text{Prob}[x\leq \alpha |Y(0)=z]\;\approx \sum_{i=1}^{z}\left(\begin{array}{c} z\\ i \end{array}\right){\left(1-\delta \right)}^{i}\delta^{z-i}(1-{\left(1-\alpha \right)}^{i})$$ (11)

$$=1-{\left(1-\alpha +\delta \alpha \right)}^{z}$$ (12)

Probability density function for the fraction of mutants, the first of which appeared when there were *z* other cells in the population is

$$f_{z}\left(\alpha \right)={\left(\text{Prob}[x\leq \alpha |Y\left(0\right)=z]\right)}^{\prime }$$ (13)

$$=\left(1-\delta \right)z{\left(1-\alpha +\delta \alpha \right)}^{z-1}$$ (14)

Then the mean fraction that the mutant will eventually achieve in the population is

$$E\left(x\right)=\delta^{z}+\int_{0}^{1}\alpha f_{z}\left(\alpha \right)d\alpha =\frac{1-\delta^{z+1}}{\left(1-\delta )(1+z\right)}$$ (15)

We can obtain the maximum likelihood (ML) estimate for the number of cells that were present in the population when the mutation that is present at a fraction *α* (for *α* < 1) appeared, by maximizing the probability distribution for the mutant fraction *f*_*z*_(*α*) Eq (14):

$${\hat{z}}_{\text{ML}}=-\frac{1}{\log[1-\alpha +\delta \alpha ]}$$ (16)

To calculate the maximum a posteriori (MAP) estimate for the number of cells that were present in the population when the mutation that is present at a fraction *α* appeared, we let *v* be the probability that a particular single mutation appears during cell division. Then *v* is also the probability that this mutation apperas in the population when there are *z* cells and forms a surviving lineage, for all *z* ≥ 1. We note that *v* is very small and on the order of 10^−9^. The probability that the successful mutation first appeared when there were *z* cells is *p*(*z*) = *v*(1−*v*)^*z*−1^ so to get the MAP estimate we will maximize *p*(*z*)*f*_*z*_(*α*):

$${\hat{z}}_{\text{MAP}}=-\frac{1}{\log[1-v]+\log[1-\alpha +\delta \alpha ]}\approx -\frac{1}{\log[1-\alpha +\delta \alpha ]}$$ (17)

since *v* is very small.

### Probability of fixation of *k*-th mutation

In a pure birth process (with *d* = 0) the founding population (type-0 and no other mutations) will always be present in the population, and thus all mutations will be present in less than 100% of tumor cells. However, when death rate *d* > 0, new mutations appearing during clonal expansion can reach fixation in the population. Probability that the *k*-th mutation with surviving lineage eventually fixates and becomes present in all cells is approximately given by

$$\rho_{k}\approx \int_{0}^{\infty }\frac{{\left(zu\right)}^{k-1}e^{-zu}u}{(k-1)!}\delta^{z}dz.$$ (18)

Here we use the fact that the population sizes at which mutations with surviving lineage appear can be approximated by a Poisson process on [0,*M*] with rate *u*[14], that final population size *M* is large and that if *k*-th mutation is produced when there are *z* other cells in the population, it will reach fixation if and only if the lineages of the other *z* cells die out. Evaluating the integral above we obtain

$$\rho_{k}\approx {\left(\frac{u}{u-\log\delta }\right)}^{k}.$$ (19)

### Fraction of cells with *k*-th mutation

Having already characterized the probability that the *k*-th passenger mutation reaches fixation in the population, we will now investigate the size of the population with the *k*-th mutation when it is subclonal.

We can derive the cumulative distribution function for the fraction of cells with the *k*-th mutation by again using the fact that the population sizes at which mutations with surviving lineage appear can be approximated via a Poisson process on [0, *M*] with rate *u*, where *M* is the final population size [14], together with result Eq (12).

$$\text{Prob}\left[x_{k}\leq \alpha \right]\approx \int_{0}^{\infty }\frac{{\left(zu\right)}^{k-1}e^{-zu}u}{\left(k-1\right)!}\left[1-{\left(1-\alpha +\delta \alpha \right)}^{z}\right]dz$$ (20)

$$=1-{\left(1-\frac{\text{log}\left(1+\left(-1+\delta \right)\alpha \right)}{u}\right)}^{-k}$$ (21)

From here we can derive the median fraction of cells with the *k*-th mutation

$$\text{Med}\left(x_{k}\right)=\text{min}\left\{1,\frac{1-e^{u(1-2^{\frac{1}{k}})}}{1-\delta }\right\}$$ (22)

### Trees

In addition to the numbers of cells carrying specific mutations, we will also investigate the phylogenetic relationships between neutral mutations in tumors. We will show that the likelihood of a particular configuration depends on the parameters on the process.

We first calculate the probability that mutation 2 appears in the lineage of mutation 1 (and not 0). We will use the approximation that the probability that mutation 2 is offspring of mutation 1 is equal to the eventual fraction of cells with mutation 1 in the population. Then the probability that mutation 2 appears in the lineage of mutation 1 is

$$p_{1\to 2}\approx E\left(x_{1}\right)=\rho_{1}+\int_{0}^{1}\alpha g_{1}\left(\alpha \right)d\alpha ,$$ (23)

where *g*_1_ is the probability distribution function for the fraction of cells with the first mutation. In other words, *g*_1_ is the derivative of the cumulative distribution function given in Eq (21) for *k* = 1. For *b* = 0.25, *d* = 0.18 and *u* = 0.015, *p*_1 → 2_ = 0.15, while for *d* = 0.2475 and *b* and *u* same as before, *p*_1 → 2_ = 0.77, in excellent agreement with simulations results.

Next we want to estimate the probabilities of each of the six trees (Fig 3) involving the first three (successful) passenger mutations. For these calculations we will need the following two quantities, sub_1_ and sub_2_, mean fractions of cells with the first and the second mutation, conditioned on their subclonality. Mean fraction of cells with the first mutation, conditioned on that mutation being subclonal, is simply

$${\text{sub}}_{1}=\frac{\int_{0}^{1}\alpha g_{1}\left(\alpha \right)d\alpha }{1-\rho_{1}}.$$ (24)

On the other hand, mean fraction of cells with the second mutation, conditioned on that mutation being subclonal, is

$${\text{sub}}_{2}=\frac{\int_{0}^{1}\alpha g_{2}\left(\alpha \right)d\alpha }{1-\rho_{2}},$$ (25)

where *g*_2_ is the derivative of the cumulative distribution function given in Eq (21) for *k* = 2.

We will also need ${\text{sub}}_{2}^{1c}$, mean fraction of cells with mutation 2, conditioned on 1 being clonal and 2 not being clonal, and ${\text{sub}}_{2}^{1nc}$, mean fraction of cells with mutation 2, conditioned on 1 not being clonal and 2 not being clonal. ${\text{sub}}_{2}^{1c}={\text{sub}}_{1}$ and since

$$E\left(x_{2}\right)=\rho_{1}^{2}+\left(1-\rho_{1}^{2}\right){\text{sub}}_{2}=\rho_{1}^{2}+\rho_{1}\left(1-\rho_{1}\right){\text{sub}}_{2}^{1c}+\left(1-\rho_{1}\right){\text{sub}}_{2}^{1nc}$$ (26)

we have

$${\text{sub}}_{2}^{1nc}=\left(1+\rho_{1}\right){\text{sub}}_{2}-\rho_{1}{\text{sub}}_{1}.$$ (27)

We will start with calculating the probability of tree 2, *p*_2_, in which mutation 2 is not offspring of 1 (event A) and mutation 3 is offspring of 1 (event B). We will denote the event that mutation 1 does not fix as C. Then *A* ⊂ *C* and

$$\begin{array}{ll} p_{2} & =P(A\cap B)=P(A\cap B\cap C)\\ & =P(C)P(A|C)P(B|A\cap C)\\ & \approx P(C)P(A|C)P(B|C) \end{array}$$ (28)

In other words, we approximate *P*(*B*|*A*∩*C*) with *P*(*B*|*C*) and obtain

$$p_{2}\approx \left(1-\rho_{1}\right)\left(1-{\text{sub}}_{1}\right){\text{sub}}_{1}.$$ (29)

Thus tree 2 occurs only when mutation 1 is subclonal (which occurs with probability 1 − *ρ*_1_), mutation 2 is offspring of mutation 0 (which occurs with probability 1 − sub_1_) and mutation 3 is offspring of mutation 1 (which occurs with probability ≈ sub_1_). When calculating the probabilities of individual trees, we again use the approximation that the probability that e.g. mutation 2 is offspring of mutation 1 is equal to the eventual fraction of cells with mutation 1 in the population.

Similarly, the probability of tree 4, *p*_4_, in which mutation 2 is offspring of 1 and mutation 3 is offspring of 0 is given by

$$p_{4}\approx \left(1-\rho_{1}\right){\text{sub}}_{1}\left(1-{\text{sub}}_{1}\right)\approx p_{2}.$$ (30)

We will calculate the probability of tree 1, *p*_1_, in a similar manner. Let *A* and *C* be the same events as above and let event *B* now be that mutation 3 is not an offspring of either 1 or 2. Then

$$\begin{array}{ll} p_{1} & =P(A\cap B)=P(A\cap B\cap C)\\ & =P(C)P(A|C)P(B|A\cap C)\\ & \approx (1-\rho_{1})(1-{\text{sub}}_{1})(1-E(x_{1}|A\cap C)-E(x_{2}|A\cap C))\\ & \approx (1-\rho_{1})(1-{\text{sub}}_{1})(1-{\text{sub}}_{1}-{\text{sub}}_{2}^{1nc}) \end{array}$$ (31)

Thus we have

$$p_{1}\approx \left(1-\rho_{1}\right)\left(1-{\text{sub}}_{1}\right)\left(1-{\text{sub}}_{1}-\left(1+\rho_{1}\right){\text{sub}}_{2}+\rho_{1}{\text{sub}}_{1}\right)$$ (32)

Similarly, the probability of tree 3, *p*_3_, is

$$\begin{array}{ll} p_{3} & \approx (1-\rho_{1})(1-{\text{sub}}_{1}){\text{sub}}_{2}^{1nc}\\ & =(1-\rho_{1})(1-{\text{sub}}_{1})((1+\rho_{1}){\text{sub}}_{2}-\rho_{1}{\text{sub}}_{1}) \end{array}$$ (33)

We now turn to calculating the probability of tree 5, *p*_5_. Tree 5 can occur when mutation 1 either fixes or does not fix. Mutation 1 fixes with probability *ρ*_1_, and then mutation 2 must not fix in the population with mutation 1 (which occurs with probability 1 − *ρ*_1_) and mutation 3 must not be offspring of mutation 2 (which occurs with probability $1-{\text{sub}}_{2}^{1c}=1-{\text{sub}}_{1})$. If mutation 1 does not fix (which occurs with probability 1 − *ρ*_1_), then mutation 2 is offspring of mutation 1 with probability sub_1_, mutation 2 does not fix in the population with mutation 1 with probability 1 − *ρ*_1_ and mutation 3 is offspring of mutation 1 but not 2 with probability $\approx {\text{sub}}_{1}-{\text{sub}}_{2}^{1nc2nc1}$. Here ${\text{sub}}_{2}^{1nc2nc1}$ is the mean fraction of cells with mutation 2, conditioned on mutation 1 not being clonal and mutation 2 being offspring but not clonal in 1. We have

$$\begin{array}{ll} p_{5} & \approx \rho_{1}(1-\rho_{1})(1-{\text{sub}}_{2}^{1c})+(1-\rho_{1}){\text{sub}}_{1}(1-\rho_{1})({\text{sub}}_{1}-{\text{sub}}_{2}^{1nc2nc1})\\ & =\rho_{1}(1-\rho_{1})(1-{\text{sub}}_{1})+{\left(1-\rho_{1}\right)}^{2}{\text{sub}}_{1}({\text{sub}}_{1}-{\text{sub}}_{2}^{1nc2nc1})\\ & \approx (1-\rho_{1})[\rho_{1}(1-{\text{sub}}_{1})+(1+\rho_{1}){\text{sub}}_{1}({\text{sub}}_{1}-{\text{sub}}_{2})] \end{array}$$ (34)

In the last equality we used the fact that

$${\text{sub}}_{2}^{1nc2nc1}\approx \frac{{\text{sub}}_{2}^{1nc}-\rho_{1}{\text{sub}}_{1}}{1-\rho_{1}}.$$ (35)

Using similar reasoning and 4 scenarios: 1) mutation 2 fixes, 2) mutation 1 fixes, but mutation 2 does not, 3) mutation 1 does not fix but mutation 2 fixes in the population with mutation 1 and 4) mutation 1 does not fix, mutation 2 does not fix in the population with mutation 1 we obtain

$$\begin{array}{ll} p_{6} & \approx \rho_{2}+\rho_{1}(1-\rho_{1}){\text{sub}}_{1}+(1-\rho_{1})\rho_{1}{\left({\text{sub}}_{1}\right)}^{2}+{\left(1-\rho_{1}\right)}^{2}{\text{sub}}_{1}{\text{sub}}_{2}^{1nc2nc1}\\ & \approx {\left(\rho_{1}\right)}^{2}+\rho_{1}(1-\rho_{1}){\text{sub}}_{1}(1-{\text{sub}}_{1})+(1-\rho_{1}^{2}){\text{sub}}_{1}{\text{sub}}_{2} \end{array}$$ (36)

The comparison between our formulas and simulation results for probabilities of each tree are shown in Table 2.

**Table 2:** Likelihood of phylogenetic trees.

	*δ*	1	2	3	4	5	6
Formulas	0.72	71.1%	9.8%	3.8%	9.8%	4.5%	1.2%
	0.96	26.4%	15.2%	9.9%	15.2%	16.1%	17.2%
	0.99	5.5%	9.7%	8.2%	9.7%	15.1%	51.7%
Simulations	0.72	73.9%	7.7%	3.5%	7.5%	6.2%	1.1%
	0.96	30.7%	12.7%	8.8%	12.7%	18.1%	16.9%
	0.99	7.4%	8.9%	7.6%	9.2%	15.2%	51.2%

Probability of each of the six trees for different values of death-birth ratio *δ*. Probabilities obtained using formulas from this section and results from 10,000 (surviving) runs of the computer simulation. Parameters: birth rate *b* = 0.25 and passenger mutation rate *u* = 0.015.

### Expected number of subclonal mutations

Let *u*_*z*_ be the probability that, when there are *z* total cells in the population, a new mutation is produced that will become subclonal and present in a fraction larger than *α*. The probability that a new mutation with surviving linage is produced before going to *z* − 1 or *z* + 1 cells is *bu*/(*b* + *d*) ⋅ (1 − *d*/*b*) = *u*(1 − *δ*)/(1 + *δ*) for *z* > 1. When *z* = 1, the probability that a new mutation with surviving lineage is produced before going to 2 cells (the only option in the process without extinction) is *u* ⋅ (1 − *d*/*b*) = *u*(1 − *δ*). For all *z* > 0, probability that the newly produced mutation is subclonal is 1 − (*d*/*b*)^*z*^.

Using formula (12), probability that a subclonal mutation with surviving lineage, that appeared when there are *z* other cells, is present in a fraction larger than *α* is

$$\text{Prob}\left[x>\alpha |x<1\right]=\frac{{\left(1-\alpha +\delta \alpha \right)}^{z}-\delta^{z}}{1-\delta^{z}}$$ (37)

This leads to

$$u_{z}=u\frac{1-\delta }{1+\delta }\left({\left(1-\alpha +\delta \alpha \right)}^{z}-\delta^{z}\right)$$ (38)

for *z* > 1 and

$$u_{1}=u\left(1-\delta \right)\left({\left(1-\alpha +\delta \alpha \right)}^{1}-\delta^{1}\right)$$ (39)

We are interested in the expected value for the number of subclonal mutations with fraction larger than *α*. Let ${\bar{m}}_{k}$ be the expected value for the number of such mutations when the process starts with *k* cells. We are again only interested in the process that does not go extinct. Thus we have

$${\bar{m}}_{1}=u_{1}+{\bar{m}}_{2}$$ (40)

$${\bar{m}}_{k}=u_{k}+p{\bar{m}}_{k+1}+\left(1-p\right){\bar{m}}_{k-1},$$ (41)

for *k* > 1 and *p* = 1/(1 + *δ*). Expressing ${\bar{m}}_{1}$ in terms of ${\bar{m}}_{k}$ we get

$${\bar{m}}_{1}=u_{1}\sum_{i=0}^{k-2}{\left(\frac{1-p}{p}\right)}^{i}+\frac{u_{2}}{p}\sum_{i=0}^{k-3}{\left(\frac{1-p}{p}\right)}^{i}+\cdots +\frac{1}{p}u_{k-1}+{\bar{m}}_{k}$$ (42)

Taking the limit of the right-hand side when *k* → ∞ and noting that ${\bar{m}}_{k}\to 0$ when *k* → ∞ we get

$$\begin{array}{ll} {\bar{m}}_{1} & =u_{1}\sum_{i=0}^{\infty }{\left(\frac{1-p}{p}\right)}^{i}+\sum_{z=2}^{\infty }\frac{u_{z}}{p}\sum_{i=0}^{\infty }{\left(\frac{1-p}{p}\right)}^{i}\\ & =\left(u_{1}+\sum_{z=2}^{\infty }\frac{u_{z}}{p}\right)\sum_{i=0}^{\infty }{\left(\frac{1-p}{p}\right)}^{i}\\ & =\left(u_{1}+\sum_{z=2}^{\infty }\frac{u_{z}}{p}\right)\frac{p}{2p-1} \end{array}$$ (43)

Plugging in the expressions for *u*_*z*_ and *p* we get that the expected value for the number of subclonal mutations with fraction larger than *α* is

$$\begin{array}{ll} {\bar{m}}_{1} & =\left(\sum_{z=1}^{\infty }u(1-\delta )({\left(1-\alpha +\delta \alpha \right)}^{z}-\delta^{z})\right)\frac{1}{1-\delta }\\ & =\sum_{z=1}^{\infty }u({\left(1-\alpha +\delta \alpha \right)}^{z}-\delta^{z}) \end{array}$$ (44)

Evaluating the last sum we obtain the expected number of subclonal mutations present in a fraction larger than *α*

$${\bar{m}}_{s}={\bar{m}}_{1}=\frac{u(1-\alpha )}{(1-\delta )\alpha }$$ (45)

### Expected number of clonal mutations

Using the same reasoning as in the previous section we can calculate the expected number of clonal mutations.

$${\bar{m}}_{c}=\sum_{z=1}^{\infty }u\delta^{z}=\frac{\delta u}{1-\delta }$$ (46)

## Supporting Information

S1 Fig Predicted and observed numbers of subclonal mutations in colorectal cancers from the TCGA dataset. We fit the cumulative distribution of allele fractions (corrected for purity) in the range [.12,.25], for 42 colorectal cancer patients in the TCGA to formula (7). 16 samples with *R* ^2^ ≥ 0.9 are shown here. We note that observed cumulative function sometimes deviates from the formula for low allele fractions (0.12-0.15), which may be due to the lower power of detecting mutations at this low frequency.
(PDF)
