# Supplementary material for: Quantifying Clonal and Subclonal Passenger Mutations in Cancer Evolution
Source: PLoS Comput Biol. 2016 Feb 1;12(2):e1004731. doi: 10.1371/journal.pcbi.1004731 (PMC4734774; doi:10.1371/journal.pcbi.1004731)
Supplement: S1 Fig — We fit the cumulative distribution of allele fractions (corrected for purity) in the range [.12,.25], for 42 colorectal cancer patients in the TCGA to formula (7). 16 samples with R 2 ≥ 0.9 are shown here. We note that observed cumulative function sometimes deviates from the formula for low allele fractions (0.12-0.15), which may be due to the lower power of detecting mutations at this low frequency. (PDF) [file pcbi.1004731.s001.pdf]

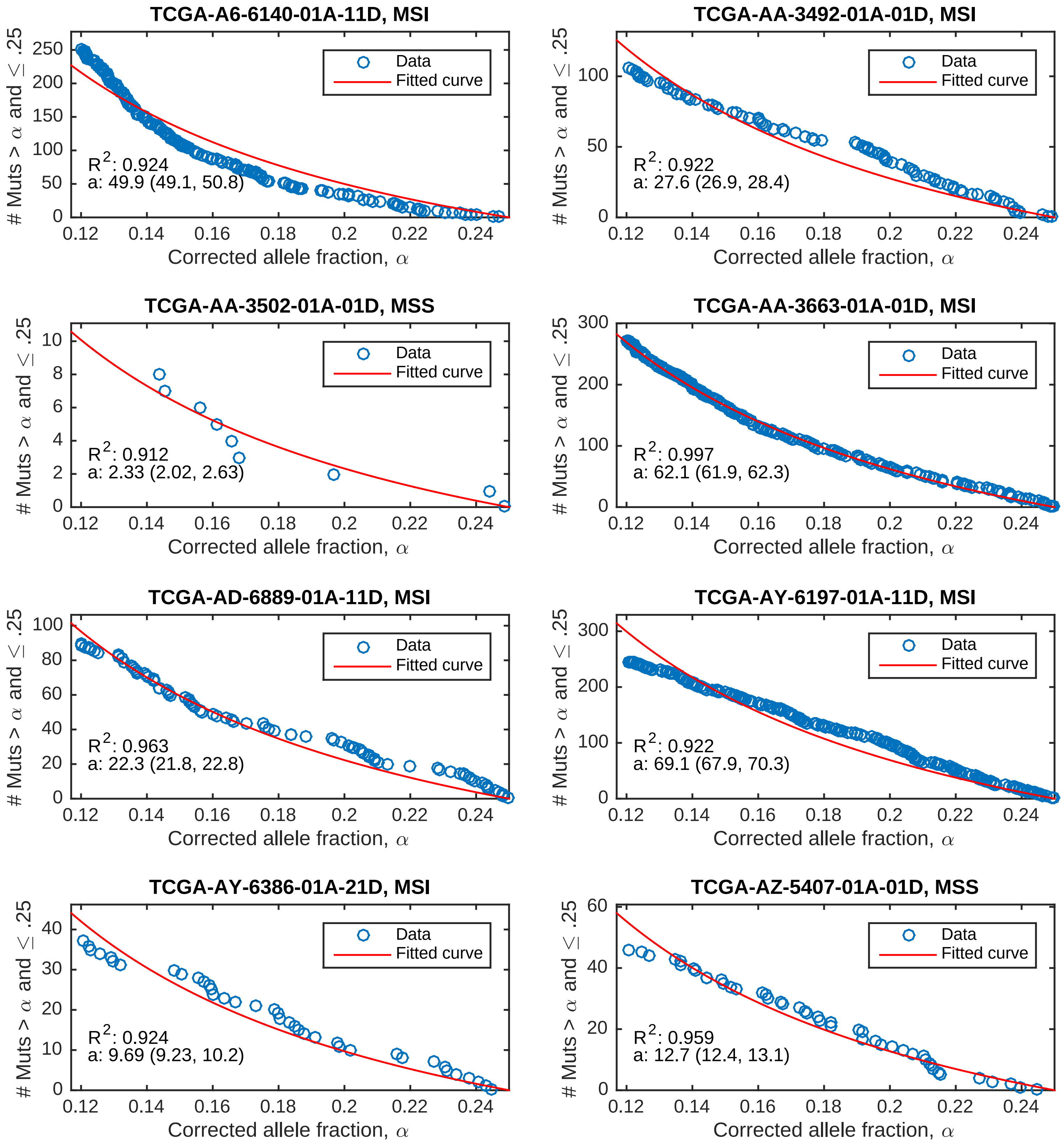
TCGA-A6-6140-01A-11D, MSI
Muts > α and ≤ .25
Corrected allele fraction, α
Data
Fitted curve
R2: 0.924
a: 49.9 (49.1, 50.8)
TCGA-AA-3492-01A-01D, MSI
R2: 0.922
a: 27.6 (26.9, 28.4)
TCGA-AA-3502-01A-01D, MSS
R2: 0.912
a: 2.33 (2.02, 2.63)
TCGA-AA-3663-01A-01D, MSI
R2: 0.997
a: 62.1 (61.9, 62.3)
TCGA-AD-6889-01A-11D, MSI
R2: 0.963
a: 22.3 (21.8, 22.8)
TCGA-AY-6197-01A-11D, MSI
R2: 0.922
a: 69.1 (67.9, 70.3)
TCGA-AY-6386-01A-21D, MSI
R2: 0.924
a: 9.69 (9.23, 10.2)
TCGA-AZ-5407-01A-01D, MSS
R2: 0.959
a: 12.7 (12.4, 13.1)
0.12
0.14
0.16
0.18
0.2
0.22
0.24

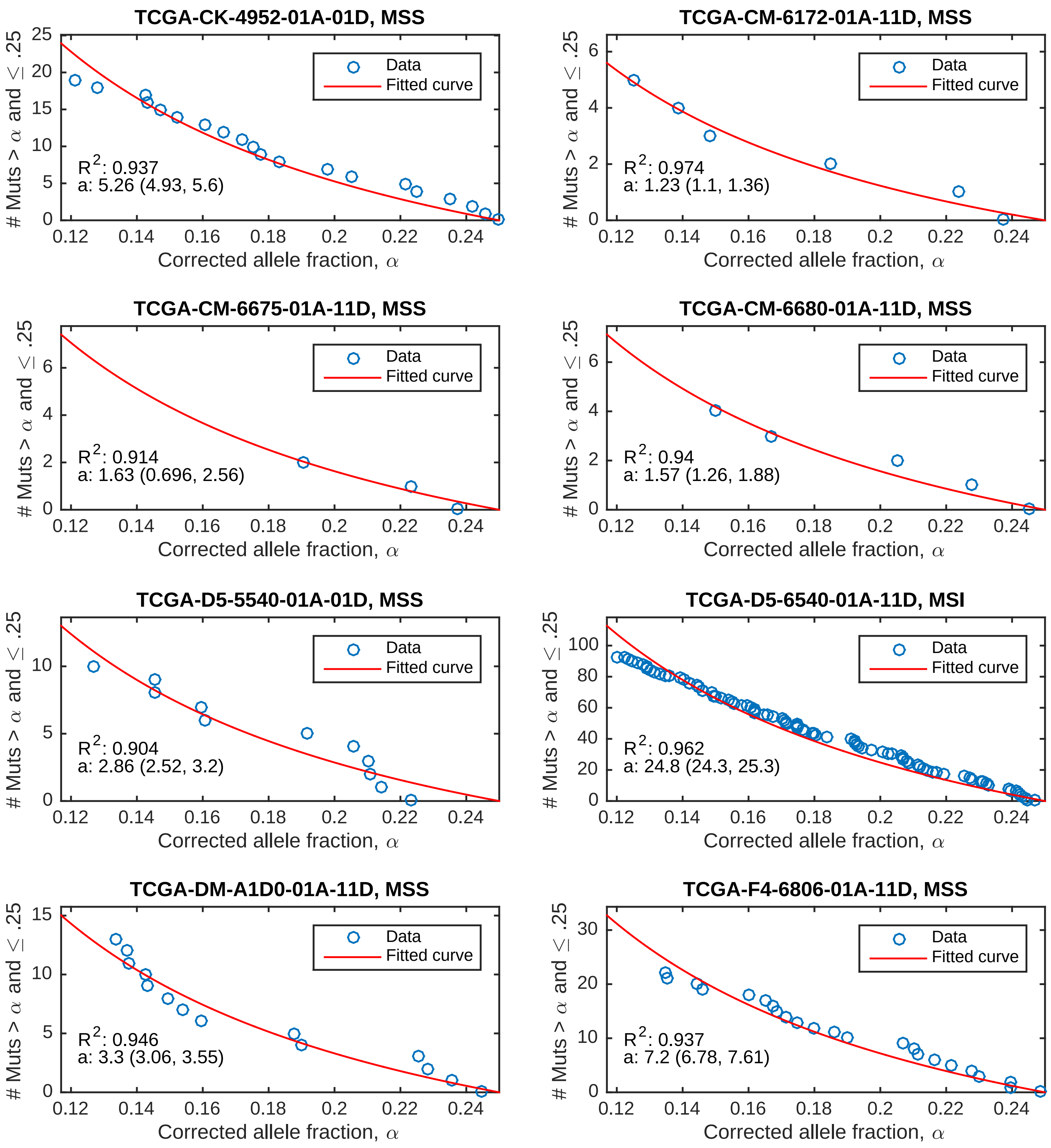
TCGA-CK-4952-01A-01D, MSS
Muts > α and ≤ .25
Corrected allele fraction, α
Data
Fitted curve
R²: 0.937
a: 5.26 (4.93, 5.6)
TCGA-CM-6172-01A-11D, MSS
Muts > α and ≤ .25
Corrected allele fraction, α
Data
Fitted curve
R²: 0.974
a: 1.23 (1.1, 1.36)
TCGA-CM-6675-01A-11D, MSS
Muts > α and ≤ .25
Corrected allele fraction, α
Data
Fitted curve
R²: 0.914
a: 1.63 (0.696, 2.56)
TCGA-CM-6680-01A-11D, MSS
Muts > α and ≤ .25
Corrected allele fraction, α
Data
Fitted curve
R²: 0.94
a: 1.57 (1.26, 1.88)
TCGA-D5-5540-01A-01D, MSS
Muts > α and ≤ .25
Corrected allele fraction, α
Data
Fitted curve
R²: 0.904
a: 2.86 (2.52, 3.2)
TCGA-D5-6540-01A-11D, MSI
Muts > α and ≤ .25
Corrected allele fraction, α
Data
Fitted curve
R²: 0.962
a: 24.8 (24.3, 25.3)
TCGA-DM-A1D0-01A-11D, MSS
Muts > α and ≤ .25
Corrected allele fraction, α
Data
Fitted curve
R²: 0.946
a: 3.3 (3.06, 3.55)
TCGA-F4-6806-01A-11D, MSS
Muts > α and ≤ .25
Corrected allele fraction, α
Data
Fitted curve
R²: 0.937
a: 7.2 (6.78, 7.61)
